# Hitting the Detection Limit in cAMP Signaling

**DOI:** 10.1093/function/zqac038

**Published:** 2022-08-18

**Authors:** Florencia Klein, Matías R Machado, Sergio Pantano

**Affiliations:** Institut Pasteur de Montevideo, Mataojo 2020, Montevideo 11400, Uruguay; Institut Pasteur de Montevideo, Mataojo 2020, Montevideo 11400, Uruguay; Institut Pasteur de Montevideo, Mataojo 2020, Montevideo 11400, Uruguay

**Keywords:** sarcomere, cyclic nucleotide, compartmentalization, concentration, subcellular localization, CUTie sensor, myofibril, nanodomain

Intracellular signaling is often conceptualized as a series of processes triggered by increasing/decreasing concentrations of small molecules. This particularly applies to the second messenger 3′,5′-cyclic adenosine monophosphate (cAMP), which regulates countless physiopathological processes. However, recent cell imaging results advocate for a paradigm change towards nanocompartmentalized intracellular events.

3′,5'-cyclic adenosine monophosphate is mainly produced from ATP upon extracellular stimulation of G protein-coupled receptors (GPCRs) that activate adenylyl cyclases. Its diffusion is limited by phosphodiesterases (PDEs), which convert it to AMP.^1^ The holotetrameric protein kinase A (PKA) constitutes a primary effector of cAMP. PKA is formed by a dimer of regulatory (R) subunits, which bind and inhibit 2 cognate catalytic (C) subunits in basal conditions. The binding of 2 cAMP molecules to cyclic nucleotide binding domains (CNBDs) present in the R-subunit triggers an allosteric transition that activates and eventually releases the C-subunits. Although PKA is a promiscuous kinase, it achieves spatial specificity by recognizing α-helical motifs in A-kinase anchoring proteins (AKAPs). Specific recognition between different AKAPs and the 4 PKA isoforms present in mammals is determined by their docking and dimerization (DD) domains located at the N-terminal of R-subunits.^2^ Unstructured peptides link the DD domains to an in-tandem pair of CNBDs. In structural terms, this implies that PKA activity may be restricted to a few tens of nanometers from a given anchoring site.^3^

Because of its biomedical relevance, the subcellular compartmentalization of cAMP has received significant attention, leading to the recent discovery of nanosized signalosome compartments.^4^,^5^ This implies that the dimensions of the nanosignalosomes' components may be critical to their function. If so, the very presence of the proteinaceous fluorescent sensors used to detect cAMP may alter the measurement by buffering it.

Recently, Anton et al. showed that individual GPCRs might constitute self-regulating cAMP nanodomains working as autonomous signaling units. Glucagon-like peptide 1 (GLP-1) and isoproterenol stimuli produce nanoconfined cAMP pools around GLP-1 and β2-adrenergic receptors, which are shielded from cAMP deriving from other cell compartments.^4^ By linking GPCRs to the so-called EPAC1-camps cAMP sensor using different “nanoruler” peptides, they showed that cAMP signals are circumscribed within 30–60 nm from the GPCR that originates them. Since the linkers used are expected to act as molecular separators without imposing a given directionality, this molecular architecture is expected to reveal cAMP within a semispherical volume of only ∼0.5 × 10^−4^ fl. This inference allows acquiring molecular-level insights into the organization of such nanosignalosomes.

Figure 1A shows a minimalist nanosignalosome, reflecting the actual dimensions of its molecular components. Considering a 10 μm concentration of cAMP and a 30 nm radius hemispherical volume, we get ∼10^−25^ moles, which gives a number of cAMP molecules within the nanodomain of about 1. This estimation is rough because we did not exclude the protein's volumes. Such limitation is unavoidable, as we ignore the identity of some components such as PDEs, phosphatases, AKAPs (which may range from a few to thousands of aminoacids), etc. However, despite the precise numerical value resulting from this simple calculation, the order of magnitude obtained implies conceptually significant issues. First, it suggests that only a handful of cAMP molecules are generated upon GPCR stimulation. Second, it calls for a distinction between intracellular concentrations and the functional presence of second messengers within the nanodomains. Noteworthy, for function *and* detection to happen, at least 3–5 cAMP molecules are needed to simultaneously activate one or both PKA R-subunits *and* the sensor. So, the sensor buffers 20%–30% of the signal in a tightly regulated regime. While this issue has been explored for other messengers such as calcium,^6^ such estimation is not available for cAMP.

**Figure 1:**
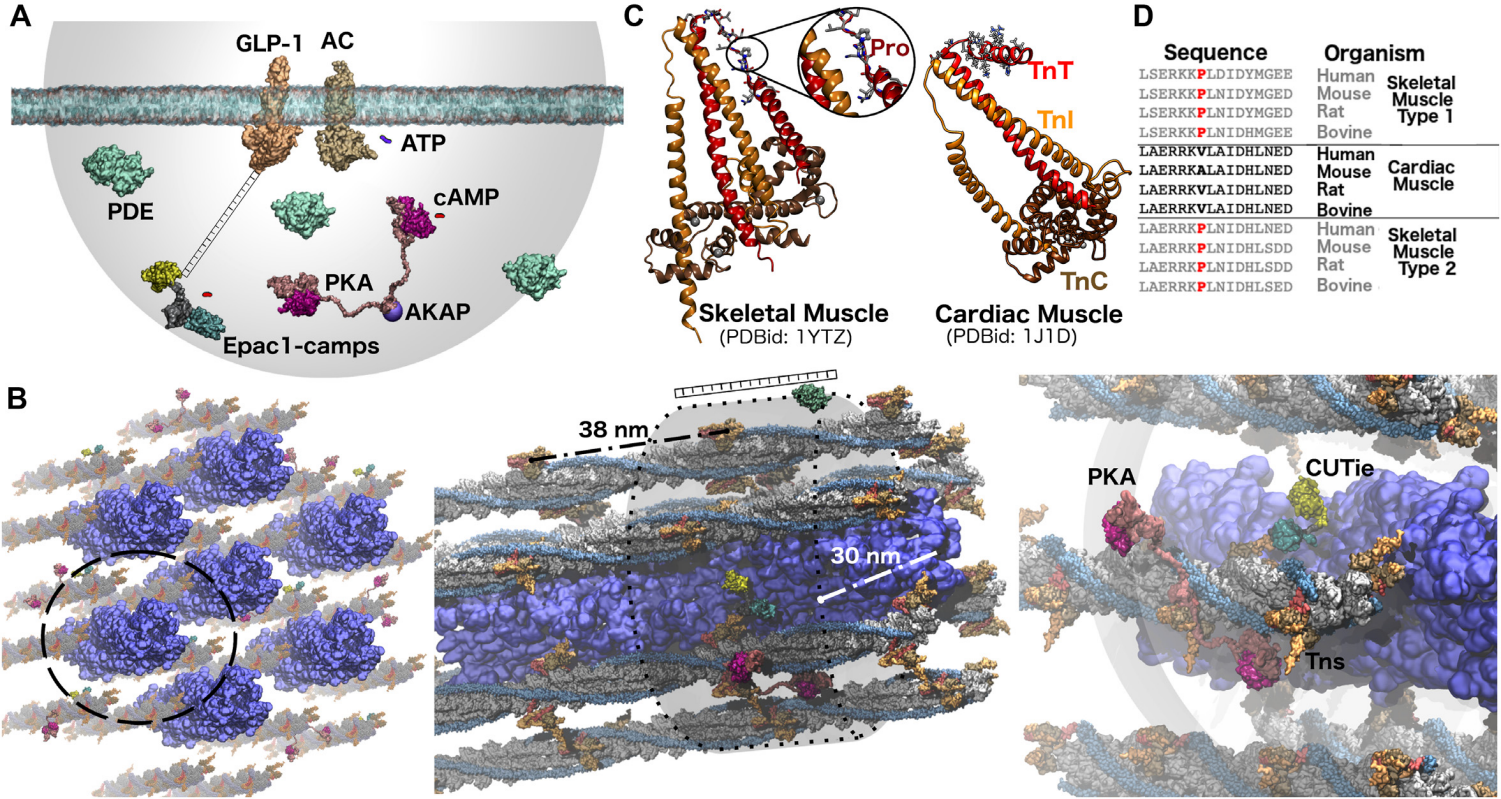
In-scale molecular representation of cAMP nanosignalosomes. (**A**) Nanosignalosome around the Glucagon-like peptide 1 (GLP-1) receptor. The structural model is based on reference 6, and all components are represented in scale relative to the 30 nm hemisphere. The model includes a membrane patch embedding the structures of the GLP-1 receptor (orange, PDBid: 6X18) and adenylyl cyclase (AC, brown, generated by AlphaFold2, https://alphafold.ebi.ac.uk/entry/P40145). The linker between the GLP-1 receptor and a model of the EPAC1-camps sensor is schematically represented as a ruler, and the semitransparent surface indicates a 30 nm radius semispherical volume. A holotetramer of PKA (PDBid: 3J4Q) with the R- and C-subunits colored pink and magenta is arbitrarily located within the nanosignalosome. The binding site of a yet undetermined AKAP is schematically indicated attached to the DD domain. The catalytic domains of 3 PDEs (PDBid:1SOJ) are arbitrarily placed near the borders of the nanodomain, just as a reference for the relative dimensions. To illustrate the size differences, ATP and cAMP molecules are placed nearby the AC and CNBDs (colored in violet and red, respectively). All molecules are represented by their solvent-accessible surface. Note that ATP and cAMP molecules occupy a comparably small but distinguishable portion of space within the nanosignalosome. (**B**) Structural model of a myofibril segment. Progressive close-ups of the myofibril are shown from left to right. The thick filament, including myosin, titin, and MyBP-C proteins, is shown in blue (based on PDB structures 3DTP and 3LPW). The volume discussed in the main text was calculated as that of a hollow cylinder, excluding the central region occupied by the thick filament. Excluding the space associated with the thin filament proteins reduces the nanosignalosome compartment to nearly half of its original value. The thin filaments are composed of actin filaments (gray) and tropomyosin (light blue), taken from the PDB structure 2W4U. Troponin systems taken from the PDB id 1J1D are structurally aligned on the previously mentioned structure of the thin filament (TnT, TnI, and TnC, red, orange, and brown, respectively). The thin filaments are aligned in a hexameric configuration centered on the thick filament, according to cryogenic electron microscopy (CryoEM) data. This structure is then replicated in space. The semitransparent cylindrical surface indicates the proposed nanosignalosome that repeats itself in space. A 30 nm ruler is added to facilitate the comparison with panel A. (**C**) Cartoon representations of skeletal and cardiac muscle troponin systems colored as in B. The protein segment proposed as AKAP in the cardiac TnT is shown with all-atoms representation, and the helix-breaking proline residue in the skeletal TnT is highlighted. (**D**) Multiple sequence alignment of the region reported as AKAP in TnT. The helix-breaking proline is highlighted in red. Structural models were built using in-house scripts and PACKMOL (http://leandro.iqm.unicamp.br/m3g/packmol/home.shtml). Protein sequences were retrieved from the Uniprot database (http://uniprot.org). Graphics were rendered using VMD (https://www.ks.uiuc.edu/Research/vmd/).

To further assess this surprising finding, we analyzed the cardiac myofilament, where we described nanometer-sized cAMP compartments using the so-called CUTie sensor fused to the regulatory protein troponin I (TnI).^5^ Using integrative modeling, we built a 3D structure including the components of the thin and thick filaments, and the CUTie-tagged troponin systems (Figure 1B). Additionally, we included a PKA bound to the AKAP motif reported on TnT.^7^ Remarkably, X-ray data reveal a helical conformation in the proposed AKAP motif, which is solvent-exposed and available for the PKA's DD domain in our model (Figure 1B and C). This helix is lost in the skeletal muscle's TnT, and sequence alignments revealed that non-cardiac TnTs present a helix-breaking Proline in this segment (Figure 1C and D). Since skeletal muscles are not subject to sympathetic regulation, this proposes the troponin system as a key component in the cardiac nanosignalosome. Because of the myofilament's repetitive structure, we can imagine it as a repetitive 3D array of independent nanosignalosomes centered on troponin systems (indicated by a semitransparent cylinder in Figure 1B).  

CryoEM data integrated into our model indicate that the troponins are separated by 38 nm along the longitudinal axis of the myofilament and 30 nm between nearby actin filaments (Figure 1B).^8^ Remarkably, this results in a volume of ∼0.6 × 10^−4^ fl, coincident with the estimation made above for GPCR systems. This might point to conserved dimensions for cAMP signalosomes in completely different structural contexts, perhaps associated with the very dimensions of the PKA holotetramer.

In this context, the fluorescent sensors would buffer a significant amount of cAMP in both cases, suggesting that current imaging setups may stress the  cell machinery. Moreover, sensors with higher/lower affinity than the endogenous cAMP target could lead to different interpretations. If cAMP is so tightly regulated, low-affinity sensors could reveal marginal or physiologically negligible signals. In contrast, high-affinity sensors could aggravate the buffering, leading to delayed cellular responses upon localized stimuli.

A possible workaround would be to perform parallel experiments with cells expressing cAMP sensors with different affinities, perhaps in combination with “cAMP sponges.”^9^ We have recently reported a simple computational protocol to design CUTie sensors with arbitrary cAMP affinities.^10^ Although technically challenging because of the few photons emitted per nanodomain, a battery of targetable CUTie sensors with different affinities could help tackle these issues helping to unravel unforeseen solutions for precision pharmacology.

## Data Availability

Data available online. The structural models in PDB format and the script used to generate the model shown in Figure 1 are available from the Zenodo Database under the DOI 10.5281/zenodo.7007705.
